# Torsades de Pointes Triggered by Transient Low‐Rate Pacing Following Leadless Pacemaker Implantation

**DOI:** 10.1002/joa3.70220

**Published:** 2025-11-12

**Authors:** Yumetsugu Munakata, Junji Morita, Yuhei Kasai, Takayuki Kitai, Yusuke Kondo

**Affiliations:** ^1^ Department of Clinical Technology Sapporo Cardiovascular Clinic Sapporo Hokkaido Japan; ^2^ Department of Cardiology Sapporo Cardiovascular Clinic Sapporo Hokkaido Japan; ^3^ Department of Cardiovascular Medicine Chiba University Graduate School of Medicine Chiba City Japan

**Keywords:** complete atrioventricular block, leadless pacemaker, torsades de pointes

## Abstract

A case of torsades de pointes triggered by transient bradycardia during Micra AV2 setup highlights a proarrhythmic risk of automated initialization. Individualized programming may be necessary in high‐risk patients with QT prolongation.

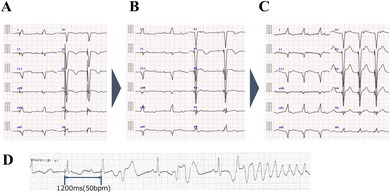

Micra AV2 (Medtronic, Minneapolis, MN, USA) is a single‐chamber right‐ventricular pacemaker that derives atrial sensing from accelerometer‐detected mechanical signals (A3/A4), thereby enabling atrioventricular (AV) synchrony. The accuracy of atrial sensing can be challenging, and optimization typically requires multiple adjustments. To facilitate this, Micra AV2 includes Atrial Sensing Setup (ASS), an automated three‐phase routine enabled by default. Phase 1 collects A3/A4 histograms for 20 min, selects the optimal A4 vector among triaxial accelerometer leads, and sets an initial A3 threshold. Phase 2 sets the A3 window end to help distinguish A3 from A4. During Phases 1–2 the device switches to VDI 50 ppm. Phase 3 returns the device to VDD and sets the A4 threshold, completing optimization (Figure 1).

**FIGURE 1:**
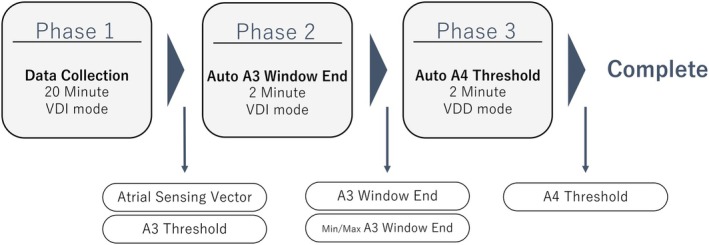
Micra AV2 Atrial Sensing Setup phases. Auto‐optimizes sensing vector, A3 window end, and A3/A4 thresholds (Phases 1–3).

An 89‐year‐old woman presented to a local hospital with syncope. ECG showed complete AV block (HR 46 bpm) with QTc 671 ms. Shortly after admission she developed torsades de pointes (TdP), which resolved with temporary VVI 70 ppm pacing. She was referred for permanent pacemaker implantation (Figure 2). On arrival, noninvasive evaluation excluded ischemic/conduction disease requiring acute therapy, and echocardiography showed preserved LVEF (50%). The patient did not receive any medications with QT‐prolonging effects. Given advanced age, dementia, and wound‐infection risk, we implanted a Micra AV2 under local anesthesia and moderate sedation in the right‐ventricular septum with stable fixation and satisfactory electrical parameters. Programming was VDD, lower rate 70 ppm, upper tracking 105 ppm, and upper sensor 120 ppm. ASS remained enabled by default at ward transfer. About 10 min later, TdP occurred on telemetry. The RR interval had prolonged to 1200 ms (≈50 bpm), implicating bradycardia (Figure 3). We immediately changed settings to VVI 90 ppm, after which no further TdP occurred. Over the next days, intrinsic rhythm improved to sinus 80–90 bpm; to limit pacing we reprogrammed to VVI 70 ppm at discharge (postimplant day 9), with no complications. Because the AV block was intermittent, discharge in VVI mode was considered appropriate.

**FIGURE 2:**
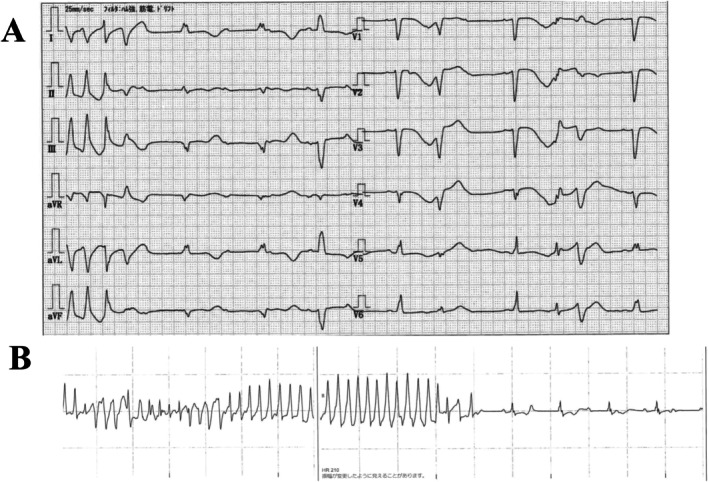
Referring‐hospital ECG. (A) ECG during torsades de pointes. (B) Pretemporary pacing ECG showing QT prolongation (QTc 671 ms).

**FIGURE 3:**
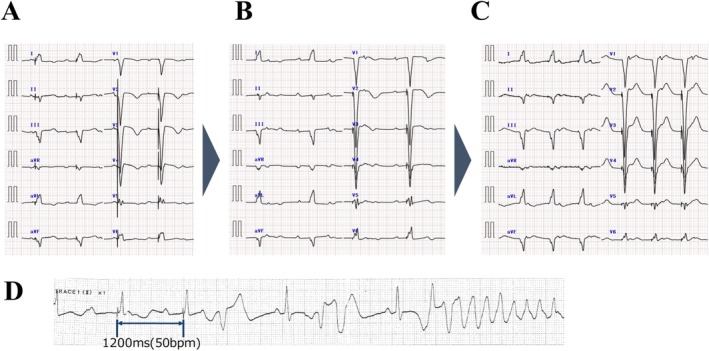
Changes in QTc on ECG. (A) Temporary pacing (VVI 70): QT 528 ms, QTc 541 ms. (B) Atrial sensing setup (VDI 50): QT 649 ms, QTc 593 ms. (C) Leadless pacing (VVI 90): QT 482 ms, QTc 529 ms. (D) Torsade de pointes during atrial sensing setup.

In this patient, Micra AV was implanted at a lower rate of 70 ppm, matching the temporary rate at the referring hospital. Because ASS was left on, the device behaved per its default routine: after telemetry, VDI 70 ppm for ~3 min, then fixed VDI 50 ppm during Phase 1 (20 min) and Phase 2 (2 min). If marked heart‐rate variability or sustained activity (> 1 min) occurs during Phases 1–2, data collection may be extended and VDI 50 ppm can persist for up to 4 h. This transient rate reduction likely precipitated TdP in our patient.

The ASS algorithm is intended to improve A3/A4 detection and generally reduces manual programming; however, the 50‐ppm limit during Phases 1–2 may pose a risk in patients vulnerable to bradycardia‐induced ventricular arrhythmias. Patients with complete AV block (CAVB) are particularly susceptible, and accumulating evidence suggests that QT prolongation in CAVB may involve mechanisms distinct from sinus bradycardia, helping explain the higher incidence of TdP at comparable heart rates.

An R‐on‐T premature ventricular contraction falling on the terminal portion of the T wave preceded TdP, suggesting pause‐dependent early afterdepolarizations (EADs) as the triggering mechanism. This finding is consistent with the concept that, in complete AV block, QT prolongation is not merely rate‐dependent but also reflects repolarization instability due to AV dissociation, including beat‐to‐beat variability and enhanced temporal dispersion of repolarization. In our case, several recognized CAVB‐related risk factors were present—advanced age, female sex, QTc 593 ms (postimplant), and a markedly prolonged Tpeak–Tend (240 ms)—which likely increased vulnerability during the ASS phase. These repolarization markers (prolonged QTc, prolonged Tpeak–Tend, LQT2‐like notched T waves, U waves/T–U fusion) have been associated with ventricular arrhythmias/TdP in CAVB cohorts [1]. Prior reports also suggest that high‐rate pacing (≈80–90 ppm) mitigates bradycardia‐related TdP; for example, maintaining ≥ 80 ppm reduced TdP in susceptible populations [2, 3]. In retrospect, programming a higher pacing rate and temporarily disabling ASS would likely have been safer, because default activation can inadvertently expose high‐risk patients to 50‐ppm bradycardia during optimization.

For patients with prior VT/VF or documented bradycardia‐related TdP, we disable ASS and program high‐rate VVI pacing (80–90 bpm) in the immediate postimplant period; once stabilized, we reassess and consider staged atrial sensing optimization.

In patients with recognized risk factors (QTc > 500 ms, Tpeak–Tend > 200 ms, LQT2‐like notched T waves, U waves/T–U fusion, low K/Mg, or use of QT‐prolonging drugs), we recommend manual atrial sensing optimization (Figure 4) [1, 4]. In the absence of these factors, routine ASS activation is reasonable, with the important caveat that the Setup process may transiently lower the effective ventricular pacing rate to 50 ppm even when the nominal lower rate is 70 ppm. Thus, strategies that transiently reduce ventricular pacing (e.g., ASS default VDI 50 ppm) must be applied with caution, as they may not reliably prevent recurrence in susceptible patients.

**FIGURE 4:**
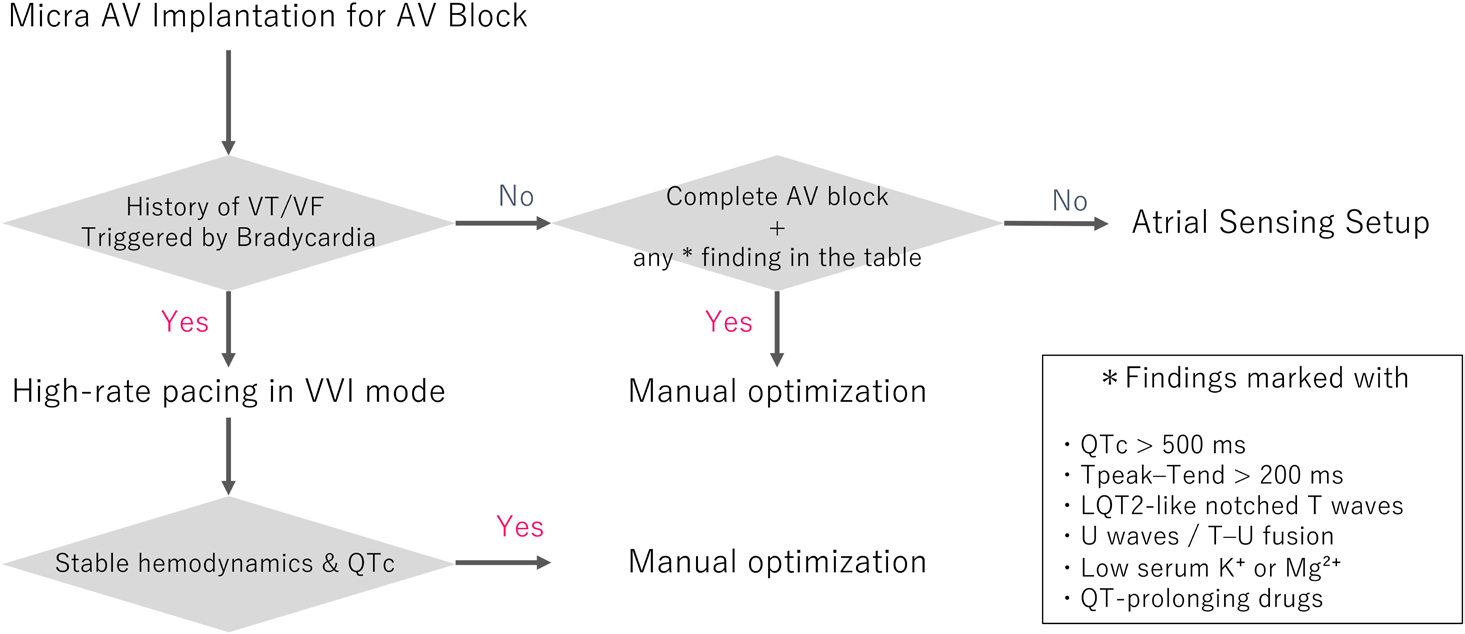
Risk stratification and management of ASS in Micra AV.

## Ethics Statement

This study was conducted according to the principles of the Declaration of Helsinki. The study was approved by the Institutional Review Board.

## Consent

The patients provided written informed consent.

## Conflicts of Interest

Dr. Kondo received lecture fees from Daiichi‐Sankyo, Medtronic Abbott Medical Japan, Biotronik, Boston Scientific, and Japan Lifeline, and research funds from Daiichi‐Sankyo and Boston Scientific. Other authors have no conflicts of interest to declare.
